# Association Between Screen Time and Lifestyle Parameters with Executive Functions in Chilean Children and Adolescents: Potential Mediating Role of Health-Related Quality of Life

**DOI:** 10.3390/children12010002

**Published:** 2024-12-24

**Authors:** Felipe Caamaño-Navarrete, Carlos Arriagada-Hernández, Lorena Jara-Tomckowiack, Jordan Hernandez-Martinez, Pablo Valdés-Badilla, Guido Contreras-Díaz, Indya del-Cuerpo, Pedro Delgado-Floody

**Affiliations:** 1 Physical Education Career, Universidad Autónoma de Chile, Temuco 4780000, Chile; felipe.caamano@uautonoma.cl (F.C.-N.); carlos.arriagada@uautonoma.cl (C.A.-H.); 2 Collaborative Research Group for School Development (GICDE), Temuco 4780000, Chile; 3 Faculty of Education, Universidad Católica de Temuco, Temuco 4780000, Chile; lorenajarat@gmail.com; 4 Department of Physical Activity Sciences, Universidad de Los Lagos, Osorno 5290000, Chile; jordan.hernandez@ulagos.cl; 5 Programa de Investigación en Deporte, Sociedad y Buen Vivir, Universidad de los Lagos, Osorno 5290000, Chile; 6 G-IDyAF Research Group, Department of Physical Activity Sciences, Universidad de Los Lagos, Osorno 5290000, Chile; 7 Department of Physical Activity Sciences, Faculty of Education Sciences, Universidad Católica del Maule, Talca 3530000, Chile; valdesbadilla@gmail.com; 8 Sports Coach Career, School of Education, Universidad Viña del Mar, Viña del Mar 2580022, Chile; 9 Escuela de Kinesiología, Facultad de Odontología y Ciencias de la Rehabilitación, Universidad San Sebastián, Lago Panguipulli 1390, Puerto Montt 5501842, Chile; guido.contreras@uss.cl; 10 Department of Physical Education and Sport, Faculty of Sports Sciences, University of Granada, 18012 Granada, Spain; delcuerpo@ugr.es; 11 Department of Physical Education, Sport and Recreation, Universidad de La Frontera, Temuco 4811230, Chile

**Keywords:** cognitive function, physical activity, schoolchildren, screen time, health-related quality of life

## Abstract

**Background/Objective**: This study aimed to (i) investigate the association between lifestyle parameters (i.e., screen time [ST], food habits, and physical activity [PA]) and health-related quality of life (HRQoL) with executive functions (EFs, i.e., attention, inhibition, working memory, and cognitive flexibility) in Chilean children and adolescents, and (ii) determine the potential mediating role of HRQoL in the relationship between ST and EFs. **Methods**: A total of 511 children and adolescents (51.3% female) aged 10–17 years participated. Lifestyle parameters and EFs were evaluated. **Results**: Attention was inversely associated with ST (β = −19.51, *p* < 0.001) and positively associated with HRQoL (β = 4.17, *p* < 0.001). Inhibition was negatively linked to ST (β = −25.17, *p* < 0.001) and positively associated with HRQoL (β = 3.23, *p* = 0.041). Working memory was inversely related to ST (β = −28.89, *p* = 0.001) and positively associated with PA (β = 34.01, *p* < 0.001) and HRQoL (β = 4.22, *p* = 0.003). Cognitive flexibility was associated with ST (β = −26.76, *p* = 0.001), PA (β = 23.23, *p* = 0.047), and HRQoL (β = 4.91, *p* = 0.004). The indirect effect confirmed that HRQoL partially mediated the relationship between ST and EFs, including attention (5%), inhibition (3.18%), working memory (3.82%), and cognitive flexibility (5.3%). **Conclusions**: ST was inversely associated with all EFs assessed, and HRQoL showed a potential mediating role in these relationships.

## 1. Introduction

Childhood and adolescence are critical periods for cognitive development, particularly for executive functions (EFs) [1]. EFs are high-level cognitive processes primarily associated with the prefrontal cortex, enabling attention, self-regulation, and goal-directed behavior [2,3,4]. These processes play a central role in learning, decision-making, and academic success [5]. Furthermore, EFs are essential for various aspects of life, including mental and physical health, as well as academic and overall success [4]. Similarly, EFs have been identified as strong predictors of students’ academic achievement [6]. Among the core EFs, inhibitory control, working memory, and cognitive flexibility are the primary focus of scientific research in this context [6].

In recent years, screen time (ST) has increased significantly among children and adolescents, encompassing activities such as watching television, using mobile phones, and playing video games [7]. Evidence increasingly links excessive ST with adverse physical, psychological, social, and neurological outcomes [8]. For example, one study demonstrated that sedentary behaviors like ST negatively impact brain structure and intelligence [9]. Excessive ST has also been shown to impair EFs and academic performance in young people [10]. A systematic review of cross-sectional and longitudinal studies found that excessive ST is negatively associated with EFs in children and adolescents [11]. Moreover, a two-year follow-up study reported that prolonged ST has a long-term negative impact on the neuropsychological development of preadolescent schoolchildren [12]. A recent narrative review further emphasized the detrimental relationship between ST and cognitive function [13]. On the other hand, adherence to ST guidelines is associated with better EF outcomes in schoolchildren [14].

Conversely, physical activity (PA) is a strong, modifiable factor that can positively influence brain function [15]. PA has been associated with various dimensions of cognitive development, including EFs, which are particularly relevant for schoolchildren [16]. Complementary research suggests that lifestyle factors, such as PA, are closely linked to EFs and the ability to learn in school settings [17]. Numerous studies have shown that PA enhances EFs [18], whereas physical inactivity negatively affects these cognitive processes [17,19]. Additionally, research indicates that low ST combined with high levels of PA is associated with better EF development [20]. Therefore, insufficient PA represents a significant risk to cognitive health.

Promoting subjective well-being and health-related quality of life (HRQoL) among children and adolescents remains a critical public health challenge [21]. The World Health Organization (WHO) defines quality of life as “individuals” perception of their position in life in the context of the culture and value systems in which they live and in relation to their goals, expectations, standards, and concerns” [22]. HRQoL is recognized as a key measure for the young population [23]. Studies have shown a positive association between EFs and HRQoL [24]. For instance, one study conducted among schoolchildren found that HRQoL indirectly affects cognitive functions [25].

However, limited information is available regarding the potential mediating role of HRQoL in the relationship between ST and EFs. Therefore, the present study aims to (i) investigate the associations between lifestyle parameters (i.e., ST, food habits, and PA), HRQoL, and EFs (i.e., attention, inhibition, working memory, and cognitive flexibility) in Chilean children and adolescents, and (ii) determine whether HRQoL mediates the relationship between ST and EFs. This study hypothesizes that increased ST is inversely associated with EFs, and that this relationship is mediated by HRQoL.

## 2. Methods

### 2.1. Participants

The present study employed a quantitative, cross-sectional, and descriptive-associative design. A total of 511 Chilean children and adolescents aged 10 to 17 years (mean age 13.68 ± 1.65 years) from Temuco, Chile, participated in this cross-sectional study (male, n = 249; female, n = 262). A total of 53 students were excluded, including 30 females and 23 males who did not meet the inclusion criteria or were excluded for other reasons. The sample was intentional and non-probabilistic.

The sample size was calculated considering the following factors: (1) enrollment of students in educational institutions appropriate for their age group (10–17 years), (2) a significance level of 5%, (3) an absolute precision of 5%, (4) a statistical power of 95%, (5) the statistical test (*T*-test), (6) the number of measurements (x1), and (7) an effect size of 0.2. Based on these parameters and accounting for an expected response rate of 80%, a sample size of 400 participants aged 10 to 17 years was determined.

The Inclusion criteria were the following: (i) participants had to be enrolled in school and (ii) be aged between 10 and 17 years. The exclusion criteria included the following: (i) any medical contraindications that would prevent normal performance in the assessments and (ii) absence during the assessment period.

The research adhered to the principles outlined in the Declaration of Helsinki, year 2013, and was approved by the Ethics Committee (Universidad Autónoma de Chile) with the approval number CEC 11-23. Participation in this study required signed assent from the schoolchildren themselves as well as informed consent from their parents or guardians.

### 2.2. Main Outcomes

#### 2.2.1. Lifestyle

A Krece Plus instrument was used to assess the students’ eating habits [26]. This tool consists of 16 dichotomous questions, which must be answered affirmatively or negatively (yes/no). Each item is scored as either +1 or −1 based on the established guidelines. The total score was classified according to previous recommendations as follows: (i) 8 to 12 points indicate optimal adherence to a Mediterranean diet (MD), (ii) 4 to 7 points suggest a need for improvement in eating habits, and (iii) 0 to 3 points reflect a diet of very low quality [26]. The Krece Plus instrument has been previously validated and used with Chilean students [27].

Physical activity (PA) was measured by asking participants how many hours per week they spent engaging in physical activity, following the recommendations from previous studies [28]. The results for PA were recorded and quantified in hours per week. Screen time (ST) was assessed using the following questions: “How many hours a week do you watch videos?” and “How many hours a week do you play video or computer games?” [26].

#### 2.2.2. Executive Function

To evaluate executive functions (EFs), including inhibition, working memory, cognitive flexibility, and attention, the CogniFit neurocognitive assessment battery (San Francisco, CA, USA) was used [29]. This 40 min assessment provides both a general cognitive score and specific scores for EFs. The CogniFit battery has been reported to exhibit good reliability and has been successfully used with school-aged children [30].

The neuropsychological test was administered online and required approximately 30 to 40 min to complete. At the conclusion of the assessment, a comprehensive results report was automatically generated, detailing the user’s neurocognitive profile. This cognitive profile has demonstrated high reliability, consistency, and stability in previous studies [31].

#### 2.2.3. Health-Related Quality of Life

Health-related quality of life (HRQoL) for participants was evaluated using the KIDSCREEN-10 questionnaire. KIDSCREEN-10 is a validated and widely used tool designed to monitor global HRQoL in children and adolescents aged 8 to 18 years. It comprises ten items, which include the following questions: Have you felt fit and well? Have you felt full of energy? Have you felt sad? Have you felt lonely? Have you had enough time for yourself? Have you been able to do the things that you want to do in your free time? Have your parent(s) treated you fairly? Have you had fun with your friends? Have you got on well at school? Have you been able to pay attention? [32].

Each item is answered on a five-point Likert scale, reflecting the frequency of a specific behavior or feeling (1 = never… 5 = always) or the intensity of an attitude (1 = not at all, 2 = slightly, 3 = moderately, 4 = very, 5 = extremely). Responses to negatively formulated items (questions 3 and 4) were reverse scored on a scale from 1 to 5. The raw scores were used for analysis, with higher values indicating better HRQoL [32].

### 2.3. Statistical Analysis

The data are presented as means and standard deviations (SDs). The assumptions of normality and homoscedasticity were evaluated using the Kolmogorov–Smirnov test and the Levene test, respectively. Differences between sexes were determined using Student’s *t*-tests.

To examine the associations between executive functions (EFs) and lifestyle parameters, a multivariable regression analysis was conducted, with results reported as beta coefficients (β) and their corresponding 95% confidence intervals (95% CIs). Two statistical models were applied: Model 1 was unadjusted, while Model 2 was adjusted for sex and age. A *p*-value of <0.05 was considered statistically significant.

Regression analyses were also performed to examine the mediating effect of health-related quality of life (HRQoL, denoted as M), with screen time (ST) as the independent variable (X) and EFs as the dependent variables (Y). The analysis included the calculation of the total effect (c), direct effect (c′), and indirect effect (a*b, IE) for the sample. These were computed along with their 95% confidence intervals using the PROCESS macro (version 3.3) for SPSS software version 23, applying a bootstrapping method with a resampling rate of 5000 [33]. The indirect effect was considered statistically significant if zero was not included within the 95% confidence interval.

The percentage of mediation was estimated as the proportion of the direct effect to the total effect, calculated as 1 − (c′/c)1 − (c′/c)1 − (c′/c). All statistical analyses were conducted using SPSS statistical software version 23.0 (SPSSTM Inc., Chicago, IL, USA), with the alpha level set at *p* < 0.05 for significance.

## 3. Results

The participants’ characteristics in lifestyle parameters and executive functions (EFs) according to sex are presented in Table 1. Significant differences were observed in lifestyle parameters, including food habits (male: 5.42 ± 2.61 vs. female: 4.23 ± 2.63, *p* < 0.001), screen time (ST) in hours per day (male: 3.66 ± 1.58 vs. female: 3.23 ± 1.40, *p* = 0.001), physical activity (PA) in hours per week (male: 1.94 ± 0.97 vs. female: 1.55 ± 0.92, *p* < 0.001), and health-related quality of life (HRQoL) (male: 26.55 ± 6.27 vs. female: 21.21 ± 6.34, *p* < 0.001).

Regarding executive functions, differences were also found in attention (male: 445.01 ± 148.99 vs. female: 399.14 ± 154.06, *p* = 0.001) and cognitive flexibility (male: 392.96 ± 248.50 vs. female: 313.05 ± 236.84, *p* < 0.001).

Attention showed an inverse and significant association with screen time (ST) (β = −19.51, *p* < 0.001) and a positive association with health-related quality of life (HRQoL) (β = 4.17, *p* < 0.001). Regarding executive functions (EFs), inhibition was inversely and significantly associated with ST (β = −25.17, *p* < 0.001) and positively associated with HRQoL (β = 3.23, *p* = 0.041).

Working memory was inversely associated with ST (β = −28.89, *p* = 0.001) and positively associated with physical activity (PA) (β = 34.01, *p* < 0.001) and HRQoL (β = 4.22, *p* = 0.003). Cognitive flexibility was significantly related to ST (β = −26.76, *p* = 0.001), PA (β = 23.23, *p* = 0.047), and HRQoL (β = 4.91, *p* = 0.004) (Table 2).

**Figure 1:**
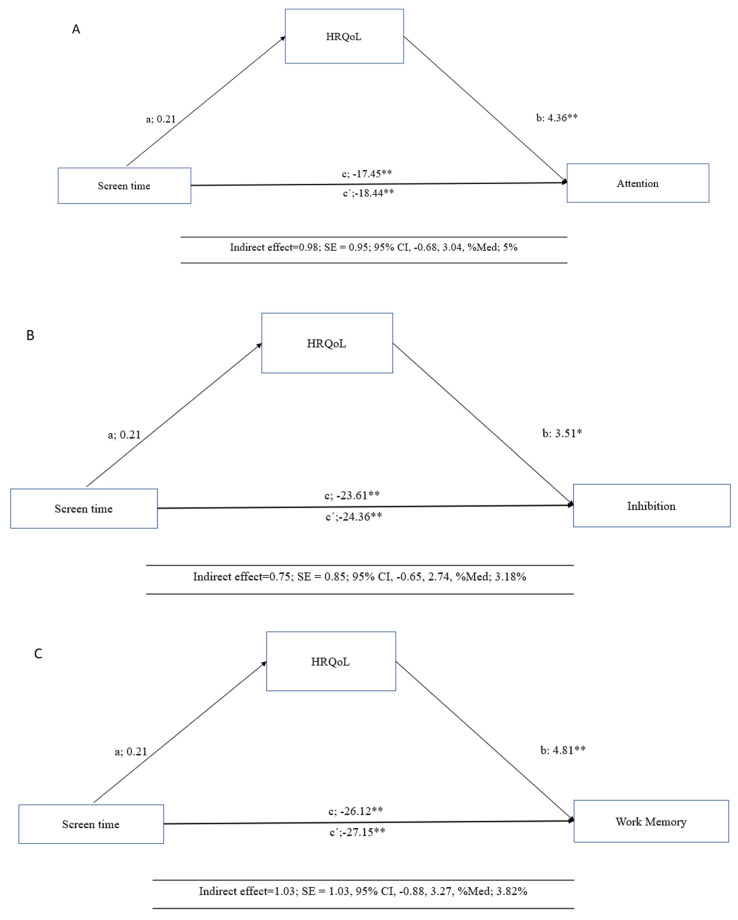

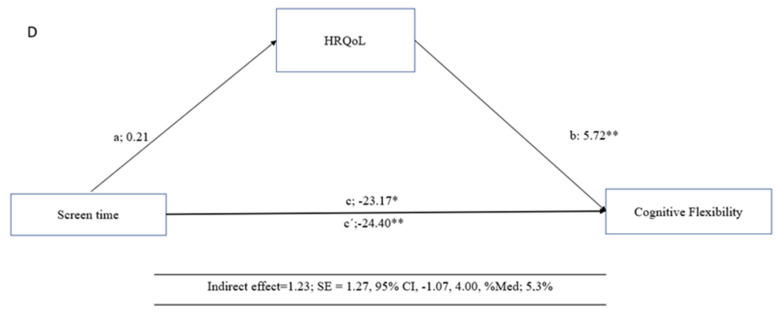
Mediation model testing whether the association between screen time with executive functions was mediated by health-related quality of life (HRQoL). * *p* < 0.05; ** *p* < 0.001. (**A**) including attention; (**B**) inhibition; (**C**) working memory; (**D**) cognitive flexibility.

The mediation analysis results are presented in Figure 1 for the total sample (n = 511). Health-related quality of life (HRQoL) emerged as a mediating variable in the relationship between screen time (ST) and executive functions (EFs), including attention (Panel A), inhibition (Panel B), working memory (Panel C), and cognitive flexibility (Panel D).

In the first regression step (a), ST was significantly associated with HRQoL (β = 0.21, *p* = 0.05). In the second step, the regression coefficients of ST for attention (β = 4.36, *p* < 0.001), inhibition (β = 3.51, *p* = 0.05), working memory (β = 4.81, *p* < 0.001), and cognitive flexibility (β = 5.72, *p* = 0.001) indicated significant associations (c′). In the third step, the potential mediator, HRQoL, was positively associated with the dependent variables of EFs (b) (*p* < 0.001). When both ST and HRQoL were included in the model (c), the regression coefficients remained statistically significant across all outcomes (*p* < 0.001).

Finally, the indirect effect confirmed that HRQoL partially mediated the relationship between ST and EFs. Specifically, the mediation effects were as follows: attention (indirect effect = 0.98; SE = 0.95; 95% CI = −0.68, 3.04, %Med; 5%), inhibition (indirect effect = 0.75; SE = 0.85; 95% CI = −0.65, 2.74, %Med; 3.18%), work memory (indirect effect = 1.03; SE = 1.03, 95% CI = −0.88, 3.27, %Med; 3.82%) and cognitive flexibility (indirect effect = 1.23; SE = 1.27, 95% CI = −1.07, 4.00, %Med; 5.3%).

## 4. Discussion

The main findings of this investigation were as follows: (i) attention was significantly associated with screen time (ST) (β = −19.51, *p* < 0.001) and health-related quality of life (HRQoL) (β = 4.17, *p* < 0.001); (ii) inhibition was inversely associated with ST (β = −25.17, *p* < 0.001) and positively associated with HRQoL (β = 3.23, *p* = 0.041); (iii) working memory was inversely related to ST (β = −28.89, *p* = 0.001) and positively associated with physical activity (PA) (β = 34.01, *p* < 0.001) and HRQoL (β = 4.22, *p* = 0.003); (iv) cognitive flexibility was significantly associated with ST (β = −26.76, *p* = 0.001), PA (β = 23.23, *p* = 0.047), and HRQoL (β = 4.91, *p* = 0.004). The mediation analysis confirmed that HRQoL partially mediated the relationship between ST and executive functions (EFs), with mediation percentages as follows: attention (%Med; 5%), inhibition (%Med; 3.18%), working memory (%Med; 3.82%), and cognitive flexibility (%Med; 5.3%).

In recent years, numerous studies have focused on examining children’s ST exposure. We found that attention, inhibition, and working memory were significantly and negatively associated with ST. Like our results, another study reported that high ST was linked with poorer EFs [34]. Complementary to the above, a cohort study indicated that meeting ST guidelines was associated with better EFs [14]. Similarly, a recent finding has shown an inverse correlation between ST exposure and EFs. In this vein, Veraksa et al. [35] studied the relationship between ST exposure and EFs, finding that in children aged 5 to 6 years, reduced ST significantly improves cognitive flexibility and inhibitory control compared to those with higher ST. In addition, our results align with those of McHarg et al. [36], who observed in a longitudinal study that ST at age 2 years is negatively associated with the development of EFs in those same children from ages 2 to 3 years. This effect is also observed in adolescents. Marciano et al. [37] emphasized in their systematic review and meta-analysis that prolonged use of digital devices is linked to lower efficiency in the cognitive control system in adolescents, particularly in brain areas related to EFs, such as working memory and cognitive flexibility. Contrary to our results, a meta-analysis indicated that ST showed no effects on EFs [38]. On the other hand, data from schoolchildren indicated a relationship between ST and EF difficulty [39]; therefore, the relationship between screen time and cognition should continue to be studied.

In this study, EFs (i.e., inhibition, working memory, and flexibility) were linked positively to health-related quality of life (HRQoL). Currently, the relationship between EFs and HRQoL has been studied, primarily in adults, highlighting the role of these cognitive skills in daily well-being and mental health. The results of published studies on this topic align with those obtained in the present study. Similarly to our results, a cross-sectional study found that poorer EFs were associated with poorer HRQoL in adolescents [40]. Complementary to the above, another study conducted in children reported that EFs were positively associated with HRQoL [41]. Similarly, another study found that lower levels of EFs were closely linked to lower HRQoL [42]. Complementary to the above, working memory was identified as a key predictor of HRQoL, suggesting that improvements in EFs could lead to a better quality of life for these young individuals. These findings are in line with those obtained in this study, but it would be interesting to compare or relate them to data from healthy populations.

In the case of adolescents, we know that self-management contributes to HRQoL during this developmental stage. Therefore, assessing EFs and health management could help identify those at risk of low HRQoL [24]. Furthermore, incorporating EF assessments as part of adolescent health measures could enhance early identification of those needing support to strengthen HRQoL. Studies suggests that focusing on cognitive skills such as working memory, inhibition, and cognitive flexibility may allow for tailored interventions that reinforce self-management abilities, ultimately fostering resilience and overall HRQoL [42,43]. Such an approach could also facilitate the development of targeted strategies in educational and clinical settings to improve mental health outcomes. Comparing findings across both healthy and clinical populations could deepen our understanding of how EFs impact HRQoL across diverse groups, emphasizing the potential benefits of early intervention and cognitive support [44].

We found that PA was associated with the EFs (working memory and flexibility). Similarly to our findings, a systematic review concluded that PA is linked with cognition [45]. Likewise, data from a meta-analysis showed a positive relation between PA and EFs in children [46]. Moreover, focusing on the field of PA, improvements in EFs—specifically in working memory and cognitive flexibility—are associated with PA. Recent studies indicate that both acute and chronic PA can have positive effects on these cognitive abilities. In this line, positive effects of PA on EFs have been reported, particularly on attention and academic performance in youth [47]. In addition, the findings by Liu et al. [48] align with those found in this study, showing that both short-term and long-term PA result in a moderate improvement in working memory and a small improvement in cognitive flexibility among youth. Additionally, focusing on children aged 8 to 12 years, it is known that higher amounts of sedentary behavior are associated with poorer EFs [49]. Furthermore, Shi et al. [50] conducted a systematic review that analyzed the impact of PA in real-world settings on EFs in typically developing children and adolescents. The results indicated that both short-term and long-term PA positively impact EFs, particularly improving working memory and cognitive flexibility when open or sequential motor skills are used. In this sense, a systematic review indicated a positive effect of physical exercise on working memory and attention [51]. These findings emphasize the importance of including physical activities in young people’s daily routines to promote the development of essential executive skills for academic achievement and adaptation in daily life.

In this study, HRQoL presented a potential mediating role in the relation between ST and EFs. Some recent studies have found that HRQoL might mediate the relationship between ST and EFs of young people. For example, with Chilean schoolchildren, it has been observed that both ST and abdominal obesity harmfully affect HRQoL, where it is shown that the quality of the muscular index mediates the relation, and some health factors could be indirectly affecting the effects of ST on HRQoL and, in turn, cognitive abilities [52].

This is further supported by the findings of a study that found certain sedentary behaviors, such as the excessive use of screen-based devices, to be related to poorer HRQoL among adolescents, possibly mediated by emotion regulation and core cognitive skills including working memory and cognitive flexibility in the context of poor general well-being [53]. Such findings underline that HRQoL is an important factor to consider in understanding the relationship between ST and EFs; therefore, improving HRQoL could buffer the negative effects of ST overuse on the cognition of children and youth.

### Limitations and Strengths

In the present study, the main limitations include its cross-sectional design, the reliance on self-reported questionnaires, and the use of a convenience sample. However, this study also has notable strengths, such as the simplicity of the assessments, which facilitates their use and application in healthy lifestyle interventions targeting children and adolescents.

## 5. Conclusions

ST was inversely associated with EFs. In contrast, lifestyle parameters, such as PA, and HRQoL showed positive and significant associations with EFs, including attention, working memory, and cognitive flexibility. Furthermore, HRQoL demonstrated a potential mediating role in the relationship between ST and EFs.

However, future research should consider employing advanced technologies to more accurately measure and assess ST.

## Figures and Tables

**Table 1:** Participants’ characteristics in lifestyle parameters and executive functions according to sex.

	Male (n = 249)	Female (n = 262)	Total (n = 511)		
				*p* Value	(f-Value)
Age (y)	13.63 ± 1.57	13.72 ± 1.71	13.68 ± 1.65	-	-
***Lifestyle Parameters***					
Food Habits (score)	5.42 ± 2.61	4.23 ± 2.63	4.81 ± 2.69	*p* < 0.001	26.42
Screen Time (h/day)	3.66 ± 1.58	3.23 ± 1.40	3.43 ± 1.50	*p* = 0.001	10.61
Physical Activity (h/week)	1.94 ± 0.97	1.55 ± 0.92	1.74 ± 0.96	*p* < 0.001	20.89
HRQoL (score)	26.55 ± 6.27	21.21 ± 6.34	23.81 ± 6.84	*p* < 0.001	91.82
***Executive Functions***					
Attention	445.01 ± 148.99	399.14 ± 154.06	421.10 ± 153.23	*p* = 0.001	11.25
Inhibition	286.80 ± 217.76	286.82 ± 235.29	286.81 ± 226.82	*p* = 0.999	0.00
Working Memory	219.18 ± 218.55	197.33 ± 197.43	207.81 ± 207.90	*p* = 0.245	1.36
Cognitive Flexibility	392.96 ± 248.50	313.05 ± 236.84	351.38 ± 245.53	*p* < 0.001	13.33

The values shown are presented as mean ± SD; *p*-value < 0.05 is considered statistically significant. HRQoL: Health-related quality of life.

**Table 2:** Association between executive function with lifestyle parameters.

Attention				
Model		β (95%CI)	Standardized Beta (SE)	*p*-Value
1	Food Habits (score)	−1.48 (−6.74; 3.78)	−0.03 (2.68)	*p* = 0.580
	Screen Time (h/day)	−19.51 (−28.36; −10.67)	−0.19 (4.50)	*p* < 0.001
	Physical Activity (h/week)	12.42 (−1.76; 26.60)	0.08 (7.22)	*p* = 0.086
	HRQoL (score)	4.17 (2.12; 6.23)	0.18 (1.05)	*p* < 0.0001
2	Food Habits (score)	−2.05 (−7.26; 3.17)	−0.04 (2.66)	*p* = 0.441
	Screen Time (h/day)	−19.50 (−28.35; −10.65)	−0.19 (4.51)	*p* < 0.000
	Physical Activity (h/week)	9.02 (−5.10; 23.14)	0.06 (7.19)	*p* = 0.210
	HRQoL (score)	3.31 (1.16; 5.46)	0.15 (1.10)	*p* = 0.003
**Inhibition**				
1	Food Habits (score)	0.81 (−7.12; 8.74)	0.01 (4.04)	*p* = 0.841
	Screen Time (h/day)	−25.17 (−38.51; −11.83)	−0.17 (6.79)	*p* < 0.001
	Physical Activity (h/week)	4.78 (−16.59; 26.16)	0.02 (10.88)	*p* = 0.660
	HRQoL (score)	3.23 (0.13; 6.33)	0.10 (1.58)	*p* = 0.041
2	Food Habits (score)	1.13 (−6.84; 9.11)	0.01 (4.06)	*p* = 0.780
	Screen Time (h/day)	−23.57 (−37.10; −10.03)	−0.16 (6.89)	*p* = 0.001
	Physical Activity (h/week)	4.64 (−16.94; 26.22)	0.02 (10.98)	*p* = 0.673
	HRQoL (score)	3.54 (0.24; 6.83)	0.11 (1.68)	*p* = 0.035
**Working memory**				
1	Food Habits (score)	−5.20 (−12.23; 1.83)	−0.07 (3.58)	*p* = 0.147
	Screen Time (h/day)	−28.89 (−40.71; −17.06)	−0.21 (6.02)	*p* < 0.001
	Physical Activity (h/week)	34.01 (15.07; 52.96)	0.16 (9.64)	*p* < 0.001
	HRQoL (score)	4.22 (1.47; 6.96)	0.141.40)	*p* = 0.003
2	Food Habits (score)	−4.98 (−11.99; 2.03)	−0.06 (3.57)	*p* = 0.164
	Screen Time (h/day)	−26.76 (−38.66; −14.86)	−0.20 (6.06)	*p* < 0.001
	Physical Activity (h/week)	32.57 (13.60; 51.53)	0.15 (9.65)	*p* = 0.001
	HRQoL (score)	4.30 (1.41; 7.20)	0.14 (1.47)	*p* = 0.004
**Cognitive flexibility**				
1	Food Habits (score)	1.47 (−7.01; 9.96)	0.02 (4.32)	*p* = 0.733
	Screen Time (h/day)	−26.07 (−40.34; −11.79)	−0.16 (7.26)	*p* < 0.001
	Physical Activity (h/week)	23.23 (0.36; 46.09)	0.09 (11.64)	*p* = 0.047
	HRQoL (score)	4.91 (1.59; 8.22)	0.14 (1.69)	*p* = 0.004
2	Food Habits (score)	0.59 (−7.66; 8.83)	0.01 (4.20)	*p* = 0.889
	Screen Time (h/day)	−24.71 (−38.70; −10.71)	−0.15 (7.12)	*p* = 0.001
	Physical Activity (h/week)	16.10 (−6.21; 38.41)	0.06 (11.35)	*p* = 0.157
	HRQoL (score)	3.37 (−0.03; 6.78)	0.09 (1.73)	*p* = 0.052

Data shown represent β (95% CI) and Standardized Beta (SE), *p*-Value. Model 1: not adjusted, Model 2; adjusted by sex and age. *p* < 0.05 denotes significant statistics. HRQoL: health-related quality of life.

## Data Availability

The data presented in this study are available on request from the corresponding author. The data are not publicly available due to Privacy.
